# Rheumatism and Gout

**Published:** 1895-08-31

**Authors:** 


### Progress in Medicine.

### RHEUMATISM AND GOUT.

(*Continued from page* 344.)

Vaughan Harley, taking the view that deposition of uric acid in urine occurs from the quadriurates being broken up by the action of the water into biurates and uric acid, argues that continuance of this action depends on the presence of sufficient acid phosphate, and this, in turn, on the conversion of $Na_2HPO_4$ into $NaH_2PO_4$ by the lecithin albumen of the kidney. If the alkaline salts are scanty, or the urates are in excess, uric acid may be precipitated even in the tubules. Thus a low percentage of chlorides, or a high degree of acidity in the urine favour early deposition.[1] On the other hand, gout would seem to depend on the retention of an abnormal proportion of uric acid salts in the blood, rather because of the failure of the kidneys than from an excessive production. Especially in those tissues where soda salts are in excess (since soda, as Roberts showed, aids in the deposition of uric acid), and where, too, the circulation is feeble, uric acid is most often deposited. Such a situation is found in the synovial membranes and cartilages. If the deposition is so rapid as to obstruct the lymph channels, a local inflammation takes place. Thus we have two distinct processes, gout or uratosis on this side of the kidney epithelium, and gravel or uratosis on the other side of it. For the latter Harley recommends piperazin, given with potash, salines, and vegetables, and especially, as suggested by Roberts, a dose of potash at bedtime to neutralise the acid urine of the night. When the production of uric acid is excessive, then alcohol, immoderate exercise, or much meat is injurious. For the digestion of meat increases leucocytosis, though uric acid is not directly derived from proteid food. Here, too, quinine and arsenic are of service by restraining leucocytosis. In gout also similar measures are useful against over production of uric acid, but above all careful treatment to aid and protect the kidneys is important. Soda salts are distinctly harmful, but the mineral springs which contain lime and magnesia, such as Contrexville, Wensidorf, and Aix-les-Bains, may be helpful. Potash is of value, but lithia and piperazin perfectly useless.[2] With respect to lithia, W. G. Smith argues that its use rests on a fallacy first because if added to serum it does not delay the deposition of uric acid, and, secondly, because it can only combine with an infinitesimal quantity of uric acid in the presence of the great amount of soda which exists in the body, since acids are partitioned among the bases present in proportion to their quantity and affinity, not to their affinity only.[3]

In a review of the anatomical changes accompanying gouty deposits Berkart notices that they are in no way peculiar to gout nor such as would be caused by the caustic action of uric acid. He insists that the deposit of urates is a result, not a cause, of the paroxysm. There is, in short, a degeneration and necrosis of tissues following on a general disease, in which myocarditis and endarteritis produce through multiplication and destruction of white corpuscles an excess of uric acid ready for deposit on the necrosed spot.[4]

Though the minute anatomy of the degenerate foci in gout and rheumatoid arthritis are similar the changes in the latter are of equal extent and in corresponding joints on the two sides, while in gout, as pointed out by E. S. Reynolds, there is no such agreement.[5] Hutchinson, in showing a patient with large tophi, mentioned that there had been nine attacks of rheumatic fever and a strong family history of gout. Indeed, he believes it is rare to find gout unaccompanied by rheumatism. He finds that gouty patients are much aided by giving plenty of bland fluids to aid elimination between the attacks. Weak tea is of especial value,[6] both in preventing gout and in aiding the elimination of uric acid. H. C. Wood speaks highly of salicylate of strontium, and Langstein of urecidin. The latter drug is said to be palatable, cheap, markedly diuretic, without irritating effects on the stomach or heart. It lessens the pain notably and removes uric acid from the system more certainly, though not always more rapidly, than any other remedy.[7] For osteoarthritis Waldo recommends fairly good living, with cod-liver oil and arsenic, and hypodermics of strychnia. The muscular atrophy chiefly affects the extensors, and is nearly always on the proximal side of the affected joint. A curious point is that this atrophy is generally accompanied by an increase in myotatic irritability; thus there may be excessive plantar reflex, foot clonus, and knee jerk.[8] Lysidin has been put forward as a solvent of uric acid of great activity, but in urine it is inert. Mendelsohn showed that this depends on the presence of an excess of sodium chloride. In the blood serum, where there is a much smaller proportion of the chloride, he found that both lysidin and piperazin easily dissolved uric acid crystals.[9]

[1] B. M. J., March 23, 1895. [2] Med. Rec., April 6. [3] Dublin J. Med. Sc., Jan. [4] B. M. J., Feb. 2. [5] Lanc., Jan. 26. [6] Clinic. J., Feb. 20. [7] Am. J. Med. Sc., March. [8] Bristol Med. C. J., June. [9] B. M. J., August 3.

